# INSIDE Project: Individual Air Pollution Exposure, Extracellular Vesicles Signaling and Hypertensive Disorder Development in Pregnancy

**DOI:** 10.3390/ijerph17239046

**Published:** 2020-12-04

**Authors:** Luca Ferrari, Francesca Borghi, Simona Iodice, Dolores Catelan, Stefano Rossi, Ilaria Giusti, Laura Grisotto, Sabrina Rovelli, Andrea Spinazzè, Rossella Alinovi, Silvana Pinelli, Laura Cantone, Laura Dioni, Benedetta Ischia, Irene Rota, Jacopo Mariani, Federica Rota, Mirjam Hoxha, Giorgia Stoppa, Damiano Monticelli, Domenico Cavallo, Enrico Bergamaschi, Marco Vicenzi, Nicola Persico, Annibale Biggeri, Andrea Cattaneo, Vincenza Dolo, Michele Miragoli, Paola Mozzoni, Valentina Bollati

**Affiliations:** 1 EPIGET LAB, Department of Clinical Sciences and Community Health, Università degli Studi di Milano, 20122 Milan, Italy; luca.ferrari@unimi.it (L.F.); simona.iodice@unimi.it (S.I.); laura.cantone@unimi.it (L.C.); laura.dioni@unimi.it (L.D.); jacopo.mariani@unimi.it (J.M.); federica.rota@unimi.it (F.R.); mirjam.hoxha@unimi.it (M.H.); 2 Department of Science and High Technology, Università degli Studi dell’Insubria, 22100 Como, Italy; f.borghi2@uninsubria.it (F.B.); sabrina.rovelli@uninsubria.it (S.R.); andrea.spinazze@uninsubria.it (A.S.); damiano.monticelli@uninsubria.it (D.M.); domenico.cavallo@uninsubria.it (D.C.); andrea.cattaneo@uninsubria.it (A.C.); 3 Department of Statistics, Computer Science and Applications “G. Parenti”, Università degli Studi di Firenze, 50134 Florence, Italy; dolores.catelan@unifi.it (D.C.); laura.grisotto@unifi.it (L.G.); giorgia.stoppa@unifi.it (G.S.); annibale.biggeri@unifi.it (A.B.); 4 Center of Excellence for Toxicological Research, Department of Medicine and Surgery, Università degli Studi di Parma, 43121 Parma, Italy; stefano.rossi@unipr.it (S.R.); rossella.alinovi@unipr.it (R.A.); silvana.pinelli@unipr.it (S.P.); enrico.bergamaschi@unito.it (E.B.); michele.miragoli@unipr.it (M.M.); paola.mozzoni@unipr.it (P.M.); 5 Department of Life, Health and Environmental Sciences, Università degli Studi dell’Aquila, 67100 L’Aquila, Italy; ilaria.giusti@univaq.it (I.G.); vincenza.dolo@univaq.it (V.D.); 6 Department of Obstetrics and Gynecology ‘L. Mangiagalli’, Fondazione IRCCS Ca’ Granda Ospedale Maggiore Policlinico, 20122 Milan, Italy; benedetta.ischia@policlinico.mi.it (B.I.); nicola.persico@unimi.it (N.P.); 7 Cardiovascular Disease Unit, Department of Internal Medicine, Fondazione IRCCS Ca’ Granda Ospedale Maggiore Policlinico, 20122 Milan, Italy; irene.rota@policlinico.mi.it (I.R.); marco.vicenzi@unimi.it (M.V.); 8 Department of Public Health Sciences and Pediatrics, Università di Torino, 10126 Turin, Italy; 9 Dyspnea Lab, Department of Clinical Sciences and Community Health, Università degli Studi di Milano, 20122 Milan, Italy; 10 Department of Clinical Sciences and Community Health, Università degli Studi di Milano, 20122 Milan, Italy

**Keywords:** air pollution exposure, hypertensive disorder development, pregnancy, extracellular vesicles

## Abstract

Hypertensive disorders are common complications during pregnancy (HDP) with substantial public health impact. Acute and chronic particulate matter (PM) exposure during pregnancy increases the risk of HDP, although the underlying molecular mechanisms remain unclear. Extracellular vesicles (EVs) may be the ideal candidates for mediating the effects of PM exposure in pregnancy as they are released in response to environmental stimuli. The INSIDE project aims to investigate this mechanism in pregnancy outcomes. The study population is enrolled at the Fetal Medicine Unit of Fondazione IRCCS Ca’Granda—Ospedale Maggiore Policlinico at 10–14 weeks of gestation. Exposure to PM_10_ and PM_2.5_ is assessed using the flexible air quality regional model (FARM) and Bayesian geostatistical models. Each woman provides a blood sample for EV analysis and circulating biomarker assessment. Moreover, a subgroup of recruited women (n = 85) is asked to participate in a cardiovascular screening program including a standard clinical evaluation, a non-invasive assessment of right ventricular function, and pulmonary circulation at rest and during exercise. These subjects are also asked to wear a personal particulate sampler, to measure PM_10_, PM_2.5_, and PM_1_. The INSIDE study is expected to identify the health impacts of PM exposure on pregnancy outcomes.

## 1. Introduction

Hypertensive disorders of pregnancy (HDP, including gestational hypertension, pre-eclampsia, and eclampsia) are common complications, affecting 2–10% of pregnancies, leading to a substantial public health impact [1,2]. Gestational hypertension is defined as a systolic blood pressure (SBP) of ≥140 mmHg or diastolic blood pressure (DBP) of ≥90 mmHg measured after 20 weeks of pregnancy in previously normotensive women [3]. Pre-eclampsia is characterized by hypertension in association with proteinuria/albuminuria [2]. In addition to non-negligible maternal mortality, pre-eclampsia and related conditions are a leading cause of maternal morbidity, perinatal death, and placental abruption [1].

Many health studies have demonstrated that exposure to acute [4,5,6,7,8] and chronic [9,10,11,12] particulate matter (PM) is associated with early death, particularly from cardiovascular (CV) disease [5,11,13]. There is also growing evidence that PM exposure during pregnancy is associated with an increased risk of developing HDP [14,15], due to an exaggerated systemic inflammatory response involving the CV system [16] and endothelium [2,17,18]. The causes of HDP are unclear, as are the molecular mechanisms linking PM exposure and HDP development.

At a functional level, the pulmonary vasoconstrictor effect of PM, combined with its systemic effects, might contribute towards explaining the link between exposure and a general decline in vascular distensibility [17]. Air pollution causes peripheral vascular dysfunction through endothelial vasoconstriction mediated by a reduction in nitric oxide (NO) activity and increased activation of endothelin-1 (ET-1) [18,19]. In the same way, air pollution causes pulmonary vasoconstriction, increasing pulmonary arterial pressure at rest, and during exercise [17].

Inhaled ultrafine PM might directly reach the systemic circulation from the pulmonary capillary bed, thus promoting atherothrombosis by stimulating a local inflammatory reaction and disrupting endothelial integrity [13,20]. Nonetheless, just a very slight portion of fine and ultrafine particles gathers in extrapulmonary structures [21]. Thus, evidence that fine particles actually enter and are deposited in blood vessels is lacking. Instead, the crosstalk between the lung and CV system might occur, potentially causing the observed peripheral effects of PM exposure [22,23].

Studies on the relationship between PM and health effects have generated a panel of circulating biomarkers reflecting inflammation [24], endothelial activation [25], platelet activation [26], oxidative damage to DNA and lipids, and antioxidant capacity [27,28]. Endothelial dysfunction occurs when a healthy endothelium becomes a damaged pro-coagulative, pro-inflammatory, and pro-vasoconstrictive phenotype. This event occurs early in many chronic diseases and reflects an imbalance in vascular function. During pregnancy, it is considered a key element in the development of pre-eclampsia [29].

Recent evidence produced by our research group showed that PM is able to modify the signaling of extracellular vesicles (EVs) [30,31,32]. This phenomenon represents a powerful mode of intercellular communication [33,34]. EVs are limited by a lipid bilayer and can be generated by cells, secreted into the extracellular space, and then enter biological fluids. EV membranes express adhesion molecules on their surface, favoring their capture by recipient cells. The fate of EVs after binding to the surface of recipient cells is not known. However, recent evidence indicates that EVs fuse with recipient cell membranes and deliver their content (e.g., miRNAs) directly to the cytoplasm of recipient cells, facilitating intercellular and inter-organ communication [35].

These characteristics of EVs make them ideal candidates for mediating the effects of PM exposure on pregnancy. For instance, EVs might be produced by the epithelial cells of the respiratory system, then translocate to the systemic circulation [36] where they interact with remote tissues, such as the placenta and maternal endothelium. Once here, they potentially deliver their cargo of bioactive molecules derived from the tissue of origin to target tissues, and post-transcriptionally regulate gene expression in recipient cells.

Furthermore, EVs might have a central role during pregnancy. Specifically, increasing quantities of syncytiotrophoblast-derived micro- and nanovesicles [37,38,39] are released during pathological pregnancy [38]. These EVs are shed from the syncytial epithelium (STB) of the placenta and are received by monocytes. This process stimulates the production of proinflammatory cytokines [40,41], perturbing maternal vascular endothelium. Endothelial damage precedes the overt manifestation of the disease and is associated with hypertension. In addition, elevated levels of soluble intercellular adhesion molecule-1 (sICAM-1) and circulating vascular cell adhesion molecule-1 (sVCAM-1) have been positively associated with preterm delivery independent of usual risk factors [42].

## 2. Aims and Hypothesis

**Figure 1:**
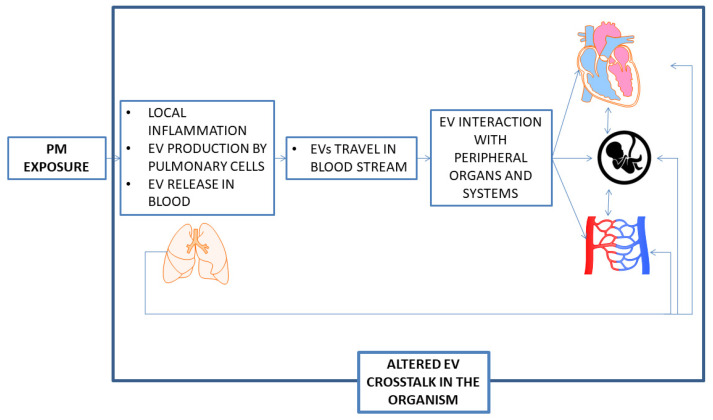
Rationale of the INSIDE project. PM: Particulate Matter; EV: Extracellular Vesicles.

The INSIDE study aims to assess the molecular effects of environmental PM exposure during pregnancy. The specific aims of this first funded INSIDE grant (PRIN 0152T74ZL_004) are summarized in Figure 1. The current study population includes 528 pregnant women (final target, n = 1000) attending the Fetal Medicine Unit (FMU) of Fondazione IRCCS Ca’Granda—Ospedale Maggiore Policlinico for routine screening for fetal chromosomal and structural abnormalities at 10–14 weeks of gestation. A subgroup of recruited women (n = 85) was asked to participate in a CV screening program, including a standard clinical evaluation (Electrocardiogram, EKG, arterial pressure measurement, evaluation of known CV risk factors) and a non-invasive assessment. The non-invasive assessment includes evaluating how right and left ventricular function and pulmonary circulation adjust during rest versus exercise.

**Figure 2:**
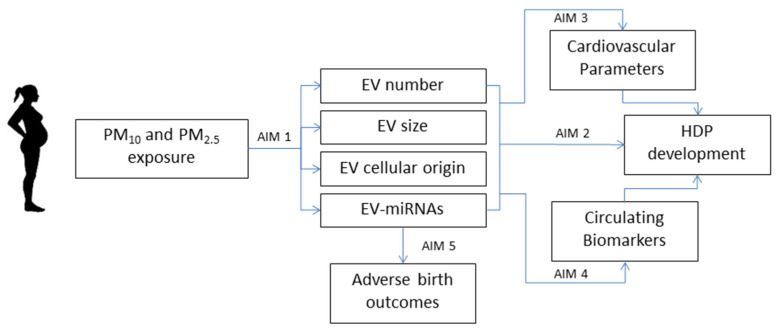
Specific aims of the INSIDE project. HDP: Hypertensive Disorders in Pegrnancy.

The study is investigating whether (Figure 2): (1) exposure to PM modifies EVs in plasma, using the quantity, size, membrane surface marker expression, and miRNA content as measures; (2) EVs are associated with the development of HDP; (3) EVs are associated with parameters collected during CV screening; (4) EVs are associated with circulating biomarkers reflecting endothelial inflammation, increased coagulation, and vascular tone, and whether these parameters could be used as indicators of subclinical effects or to predict clinical outcomes involving endothelium homeostasis; (5) EVs are associated with adverse birth outcomes, such as low birth weight, preterm birth, and perinatal morbidity. The current manuscript presents the study design, field activities, management approach, and characteristics of the enrolled women.

## 3. Methods and Analysis

### 3.1. Study Design and Cohort Description

The INSIDE study is a cross-sectional study that investigates how exposure to air pollution impacts a population of pregnant women residing in the Lombardy region, Italy. The “Clinica Mangiagalli”, Fondazione IRCCS Ca’Granda—Ospedale Maggiore Policlinico is a hospital located in Milan, Italy, which is the capital city of the Lombardy region. The “Clinica Mangiagalli” has the highest number of births in Lombardy.

The target of the INSIDE study is the enrolment of 1000 women attending the FMU for screening for fetal chromosomal and structural abnormalities at 10–14 weeks of gestation. The recruitment period started in June 2014 and is ongoing. So far, 518 subjects have been enrolled. We are currently seeking further funding to complete the accrual of 1000 subjects. The eligibility criteria for participants were: (1) older than 18 years at enrolment; (2) physiological pregnancy; (3) resident in Lombardy at the time of recruitment; and (4) agreed to sign an informed consent and donate a blood sample. Exclusion criteria included a previous diagnosis of cancer and cardiovascular diseases or other chronic diseases, such as multiple sclerosis, epilepsy, schizophrenia, and depression. The participation rate of the overall population was 76%. The main characteristics of the study participants are reported in Table 1. The mean age is 33.8 (±4.3) years and the mean gestational age at enrolment is 11.9 (±0.7) weeks. It was the first pregnancy for 60.8% of the women, with the remainder having had one or more children. The mean pre-pregnancy body mass index (BMI) is 22.4 (±3.7) kg/m².

### 3.2. Epidemiological and Clinical Data Collection

Pre-gestational maternal information (such as age, educational level, ethnicity, parity, and folic acid supplementation) are obtained using a questionnaire at enrollment. Information on place of residence, cigarette smoking, alcohol consumption, medical history, and medication are also collected. For the specific purpose of the present project, each patient is asked to: (1) sign an informed consent form explaining the study aims and procedures; and (2) provide a 15 mL peripheral blood sample. We also asked 85 enrolled patients to undergo a cardiac ultrasound and to be available for being recalled during the second (20–24 weeks) trimester of the gestational period for a cardiovascular follow-up. Repeated measurements analysis was performed at rest and after exercise to explore patterns in the cardiovascular response.

### 3.3. Cardiovascular Assessment

The clinical assessment of patients includes at rest evaluation through blood pressure, heart rate, and peripheral arterial oxygen saturation (SpO_2_). Flow-mediated dilation of the brachial artery is performed by adopting a standard approach. In brief, vessel diameter and blood flow at rest are compared with those measured 5 min after distal occlusion. We record endothelial response every 15 s over a 90 s period after the release of occlusion.

CV adaptation during effort is assessed through a cardiorespiratory exercise test (CPET) that is combined with exercise echocardiography on a semi-recumbent cycle ergometer tilted 20° to 30° to the left. Once a warm-up phase at 15 Watts ends, standardized effort with stages of 20 Watts every 2 min starts. Based on the international guidelines for the exercise test, continuous monitoring of cardiac rhythm and blood pressure is carried out during effort. Exercise is stopped when 70% of maximal heart rate is achieved (formula: 220-age of the subject). Cardiac output (Q), mean pulmonary arterial pressure (mPAP), and left atrial filling pressure (LAP) are calculated from echocardiography-derived equations. Total pulmonary vascular resistance is calculated as the mPAP/Q ratio. Pulmonary vascular adaptation to exercise is described by the slope of the multipoint mPAP–Q curve. The CPET is used to qualify the respiratory response and quantify the rise in ventilation per minute induced by exercise.

### 3.4. Measures of the Fetus

Measurements of the fetus are collected as part of the prenatal screening test, during which the fetus is examined by ultrasound and blood is drawn for the INSIDE project. During ultrasound examination, data about crown–rump length, nuchal translucency, and fetal heart rate are registered. Gestational age is calculated from the first day of the last menstrual period.

### 3.5. Outcome at Birth

Information on the evolution of fetal growth and neonatal conditions is obtained from the Certificate of Delivery Care (registry from Lombardy Region). Neonatal weight (g), neonatal length (cm), cranial circumference (cm), and Apgar score are registered.

### 3.6. Collection of Biological Samples

We developed a specific standard operating procedure to guarantee the quality control of each step involved in sample collection and analysis. Blood is collected in ethylenediaminetetraacetic acid (EDTA) tubes (7 mL), and transported to the EPIGET Lab (University of Milan) within 2 h of phlebotomy. Blood-EDTA is processed to separate buffy coat and plasma, by centrifuging at 1100× *g* for 15 min at room temperature. To obtain the EV pellet, an aliquot of plasma is further centrifuged at 1000, 2000, and 3000× *g* for 15 min at 4 °C to remove cell debris. It is then transferred to an ultracentrifuge tube (Quick-Seal-Round-Top, polypropylene, 13.5 mL, Beckman Coulter, Inc., Brea, CA, USA), and diluted with phosphate-buffered saline (PBS).

### 3.7. Investigation of Extracellular Vesicles

EVs are investigated by characterizing the determinants of several membrane determinants that are suggestive of EV cellular origin. EV size, EV count by nanoparticle tracking analysis (Nanosight NS300, Malvern Panalytical, Malvern, UK), and EV content (in particular, microRNAs) are also analyzed. For miRNA analysis, we follow a two-stage, split-sample study design. The discovery stage was conducted on the first 212 women consecutively recruited by OpenArray technology QuantStudio™ 12K Flex (Thermofisher Scientific, Waltham, MA, USA), which is a fixed-content panel containing validated human TaqMan microRNA assays derived from Sanger miRBase release v.14 [43]. The panel analysis screens 754 miRNAs and the full list of targets investigated is available online [44]. The validation stage is being conducted by standard real-time PCR on the top differential miRNAs defined at the discovery stage.

### 3.8. Biochemical Measurements

Circulating biomarkers reflecting inflammation status (IL-6), coagulation activation, and early systemic prothrombotic effects (C-reactive protein, plasma fibrinogen, plasminogen activator inhibitor-1) are evaluated, along with the functional assessment of the endothelium (circulating levels of inflammatory mediators, sICAM-1 and sVCAM-1), in human plasma samples using quantitative sandwich enzyme immunoassay techniques. Assays are performed using the Ella Automated Immunoassay System (ProteinSimple, San Jose, CA, USA) inside a patented glass nano-reactor. The entire process is controlled with microfluidics.

### 3.9. Exposure Assessment

#### 3.9.1. FARM Model

**Figure 3:**
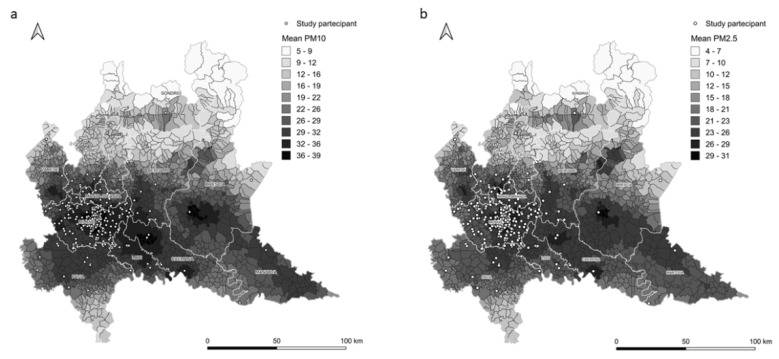
PM_10_ (2014–2019; (**a**)) and PM_2.5_ (2014–2019; (**b**)) concentration estimates at municipality resolution predicted by the flexible air quality regional model (FARM).

Daily concentrations of air pollutants (PM_10_ and PM_2.5_) are derived from the archives of the Regional Environmental Protection Agency (ARPA Lombardy). This organization collects data at a regional scale using the flexible air quality regional model (FARM) chemical–physical model of air quality [45]. This model is a three-dimensional Eulerian model that simulates the dispersion and chemical reactions of atmospheric pollutants. The system for forecasting pollutant concentrations is composed of a meteorological model powered by simulation data. In comparison, for the initial and boundary conditions, the outputs of the “Quale Aria” system are used [46]. Emissions are retrieved from regional, national, and European inventories. The domain of the simulation with the air quality FARM covers the entire Lombardy region with a grid of 1 × 1 cm^2^ cells generated by the website, with daily estimates at municipality resolution (Figure 3). Finally, concentration data measured from the stations of the ARPA air quality network are integrated in the simulation results using interpolation techniques [47]. The estimated levels of daily PM_10_ and PM_2.5_ are assigned to each subject for the day of evaluation and 90 days before blood is sampled. We also calculate the average exposure from the day of the clinic visit and 90 days earlier. All participants are assigned pollutant levels that are estimated in the place of residence and in the municipality of Milan.

#### 3.9.2. Bayesian Geostatistical Model

Using monitored data from the air quality network and the output of a photochemical deterministic model of the Environmental Protection Agency for the Lombardy region, we are currently developing a Bayesian geostatistical model to account for land use covariates, uncertainty in deterministic model outputs, and preferential sampling due to non-random monitoring locations [48,49,50].

#### 3.9.3. Personal Air Pollution Measurements

Personal exposure to PM was measured in a subset of 85 pregnant women over two consecutive winter periods (October 2017–April 2018 and October 2018–April 2019). Subjects were instructed to wear a personal sampling device to measure exposure levels continuously over a 1–6 h period before clinical evaluation. During the monitoring period, all the enrolled subjects were requested to do their routine activities. Exposure to fractionated PM (PM_1_, PM_2.5_, PM_4_, PM_10_, and TSP-Total suspended particles) was evaluated through a direct-reading monitor (Aerocet 831—Met One Instrument Inc., Grant Pass, OR, USA) set with an acquisition rate of 1 min. To improve accuracy, *a posteriori* corrections were applied to raw data based on daily comparisons with gravimetric PM_2.5_ data collected by a Harvard impactor (Diagnostic and Engineering, Inc., Harrison, ME, USA), which was used as a gold standard [51]. A mobile phone (LG K4 2017) with an Android application (Geo tracker GPS tracker; Version 3.3.0) was used to determine and record subjects’ position during the monitoring sessions. In order to make the reconstruction of the routes traveled and the visited environments more complete, the enrolled subjects were asked to fill a time activity diary.

#### 3.9.4. Sample Size

The power calculation was based on testing the association of short-term exposure with the sum of EVs using standardized regression coefficients. A sample size of 1000 pregnant women was estimated to achieve 95% power to detect a reduction in the EV slope from a 0.10 standard deviation (SD) under the null hypothesis to a 0.16 SD under the alternative hypothesis for a 1 SD change in exposure. From our preliminary analysis, we assume a correlation between PM_10_ and the sum of EVs of 0.30, and a two-sided significance level of 0.05. The same approach was used to define the sample size for the subgroup of women enrolled for the personal sample exposure measure. The sample size was based on testing the association of short-term exposure on the rest heart rate (HR). A sample size of 85 pregnant women was estimated to achieves 90% power to detect a change in the rest HR slope from a 0.10 SD under the null hypothesis to a 0.99 SD under the alternative hypothesis for a 1 SD change in exposure. From our preliminary analysis, we assume a correlation between PM_10_ and the rest HR of 0.37.

#### 3.9.5. Statistical Analysis

Descriptive statistics will be performed on all variables. Graphical inspection of the main variables of interest will be performed to examine their distribution and uncover the need for eventual transformation. Given the specific aims of the INSIDE project reported in Figure 2, we will evaluate the associations between individual and particulate matter exposure in the following outcomes: EV concentration and miRNAs, cardiovascular parameters, adverse birth outcomes, HDP parameters, and circulating biomarkers. As a second step, we will perform mediation analyses to estimate the extent to which the EV concentration and miRNAs mediate the effects of PM exposure on each specific outcome.

Multivariable modeling will be based on both biological and statistical considerations. For each outcome, we will identify a priori covariates that need to be controlled for, based on biological consideration and the current state of the literature. Other potential confounders will be included in the multivariate model after having verified the presence of an association in a univariate model. The best model selection will be based on the minimization of the Akaike information criterion and maximization of the explained variance of the model. We will conduct residual analysis to confirm model fit, identify highly influential data points, and possible nonlinear relationships between predictors and outcomes.

Finally, the best model will be selected to predict the association between PM exposure and each specific outcome.

To compare the magnitude of the association with different exposures, we will calculate standardized regression coefficients, which represent the change (standard deviation) in a dependent variable resulting from one standard deviation change in the independent variable.

Due to the high number of comparisons, we will apply a multiple comparison correction method based on the Benjamini–Hochberg false discovery rate (FDR) to calculate the FDR *p*-value.

Multivariable linear regression models will be also used to test the association between PM exposure and outcome in the mediation analysis framework, where we hypothesize that the relationship can be mediated through changes in miRNA expression, EV count, or their characterization. Each EV count and each miRNA will be separately considered as a potential mediator in a mediation model. We will estimate the direct effect and the indirect (mediated) effect with bias-corrected bootstrap confidence intervals.

All statistical analyses will be performed by using SAS 9.4 statistical software (SAS Institute Inc., Cary, NC, USA) and R software (version 3.6.1; Foundation for Statistical Computing, Vienna, Austria). Mediation analyses will be executed on the SAS v9.4 macro, using the PROCESS program provided by Bolin [52]

## 4. Ethics and Dissemination

The study design, research aims, and measurements were approved by the Ethics Committee “Comitato Etico—Milano Area 2” of the Fondazione IRCCS Ca’ Granda Ospedale Maggiore Policlinico, 20122 Milan, Italy (approval number #318), in accordance with principles of the Helsinki Declaration. Each participant signed a written informed consent form including a detailed description of the study. The measurements of new parameters will only be inserted in the study after the approval of the Ethical Committee. The datasets used and/or analyzed during the current study are available from the corresponding author on reasonable request.

All the scientific communications will be carried out under the responsibility of the study principal investigator with the agreement of the collaborators.

## 5. Data Management and Privacy Protection

The INSIDE study collects a large amount of information. To protect the privacy of each subject, all information and biological samples collected are anonymized of personal identifying information and can be identified only through a five-digit randomly assigned barcode. The link between the barcode and the subject’s identity is held in a secure database. All the data collected through the questionnaire forms are imputed in the database, and quality and completeness controls are performed weekly. All data are combined in a central relational database (MS SQL Server). Data processing is anonymous, and the highest level of confidentiality is maintained for all identifying information. We check the quality of collected data by comparing information from different sources (e.g., clinical records, molecular and biochemical exams), assessing the ranges and distributions of variables, evaluating the quality of biospecimens through specific analyses conducted on random samples, and verifying database completeness through multiple queries. The complete list of all collected variables is provided in Supplementary Materials Table S1.

## 6. Opportunities to Collaborate

We designed INSIDE due to our awareness that its value would consistently increase thanks to the involvement of other investigators, as individuals, and within consortia. The INSIDE project is open for collaboration. Given the detailed epidemiological data available, such as the clinical information and molecular data collected, INSIDE offers a good opportunity to collaborate. Proposals to test specific hypotheses on the INSIDE population that come from outside the study team will be reviewed by our research team. Requests can be sent to the e-mail valentina.bollati@unimi.it.

## 7. Discussion

The investigation of mechanisms linking the inhalation of particulate air pollutants to negative health effects is considered a pressing priority [53,54]. In particular, it is important to acquire knowledge of how air pollutants impact hypersusceptible populations, including pregnant women, who are widely documented to have high sensitivity to several toxicants and pollutants, including fine particles. Moreover, adverse effects on the wellbeing of pregnant women and their newborn children are not acceptable, given their very high social and public health impact [1].

In fact, pre-eclampsia, and consequent fetal growth restriction and preterm births, are related to acute maternal and neonatal mortality and morbidity. It is also associated with long-term compliance and social costs. For instance, pre-eclampsia decreases the future health-related quality of life of mothers and increases the risk of post-partum depression [1,2,3]. Moreover, children born to mothers with pre-eclampsia have an increased short-term risk of bronchopulmonary and cerebral complications, as well as a long-term increased risk of hypertension, diabetes, and metabolic diseases [13,55,56].

Large epidemiological studies have demonstrated adverse pregnancy outcomes associated with high levels of maternal PM exposure [57,58,59,60]. However, the molecular and biological mechanisms linking the inhalation of PM with fetal homeostasis are still lacking. Consequently, the involved mechanisms that could be potentially modified by preventive or drug-based interventions remain unknown and, hence, untreatable. However, the identification of miRNAs in the plasma of the EVs of healthy subjects potentially provides information on the potential mechanism that alters maternal blood pressure. Specifically, EVs produced by the lungs after PM stimulation might be able to transfer a specific pattern of miRNAs to other regions of the body, such as the placenta or endothelium [32,35,36].

Our multi-step approach was specifically designed to elucidate how EVs contribute to the casual pathway from maternal exposure to pregnancy outcomes (based on tissue-specific processes and functions) and is expected to generate biologically meaningful results. This study is examining these mechanisms in a large group of pregnant women, who are being recruited from the biggest and most important maternal clinic of Milan. The metropolitan area of this Italian city is characterized by one of the highest levels of PM in Europe. EV production and content (miRNAs) in plasma are measured in the recruited women at critical time points. Plasma is an easily obtainable biological medium and can be safely obtained, even from pregnant women. If we were successful in identifying changes to miRNAs in EVs, this information might be useful in clinical settings to generate potential future preventive and diagnostic applications. In addition, the miRNAs in EVs might also be a drug target, potentially opening pathways to develop future interventions to reverse the effects of air pollution in pregnant women, generating positive public health impact.

Moreover, our investigation focused on identifying molecular and biological changes before the development of a critical disease that is difficult to mitigate (maternal hypertension, pre-eclampsia, and consequent preterm delivery). It is important to identify pre-clinical effects in environmental health studies because pre-clinical effects impact a large component of the exposed population when pollutant levels are moderately low, such as in urban areas outside periods of PM peaks. This information would also allow physicians to implement effective preventative measures. Finally, it is important to assess biochemical and functional abnormalities to determine casual pathways, ranging from exposure to disease, and to elucidate critical mechanisms to help mitigate effects in pregnant women and the general population.

## 8. Conclusions

The outputs of the INSIDE project are expected to identify the health impacts of PM exposure and to determine risks to the general population, particularly the hypersusceptible sub-population of pregnant women. This information is necessary to develop evidence-based preventive health care programs. In conclusion, our results are expected to contribute to the priorities of the National Prevention Plan, which considers pollution risk prevention as a key objective.

## 9. Patient and Public Involvement

Patients and/or the public were not involved in the design, or conduct, or reporting, or dissemination plans of this research.

## Figures and Tables

**Table 1:** Characteristics of study participants.

Characteristic (n = 518)	Value
Age, mean ± SD, year	33.8 ± 4.3
Gestational age at sample, weeks	11.9 ± 0.7
**Season of Enrollment, n (%)**	
Winter	161 ± 31.1
Spring	116 ± 22.4
Summer	100 ± 19.3
Autumn	141 ± 27.2
**Anthropometric and Biochemical Features**	
BMI, Kg/m²	22.4 ± 3.7
*Categorical BMI*	
Underweight (BMI < 18.5)	50 (9.8%)
Lean (18.5 ≤ BMI < 25)	358 (69.8%)
Overweight (BMI ≥ 25)	105 (20.5%)
Glucose, mg/dL	86.5 ± 14.6
Total Cholesterol (TC,) mg/dL	181.9 ± 29.2
Low-density lipoprotein-Cholesterol (LDL-C), mg/dL	97.4 ± 22.1
Lipoprotein a, mg/dL	6 (3, 13)
non-High Density Lipoprotein-Cholesterol HDL-C, mg/dL	116.8 ± 25.0
HDL-C, mg/dL	65.5 ± 13.9
Triglycerides (TG), mg/dL	91.6 (69.6, 112.6)
Proprotein convertase subtilisin/kexin type 9 (PCSK9), ng/mL	182.6 (155.2, 224.7)
Intracellula Adhesion Molecul (ICAM), pg/mL	339,664 ± 67,973
Vascular cell adhesion protein (VCAM), pg/mL	786,866 ± 179,405
C-reactive Protein (CRP) mg/dL	2.6 (1.5, 4.6)
Fibrinogen, mg/dL	139.3 (112.2, 174.6)
IL-6, pg/mL	1.7 (1.4, 2.1)
Serpin, pg/ml	19,098 (13825, 25089)
**Smoking Habits, n (%)**	
Never smoked	435 (84.0%)
Stopped during pregnancy	55 (10.6%)
Smoker	25 (4.8%)
**Features Related to Pregnancy**	
*Parity*	
Nulliparity	315 (60.8%)
Multiparity	201 (38.8%)
*Pregnancy-associated endocrine factors*	
Pregnancy-associated plasma Protein A (PAPPA), IU/L, median (Q1, Q3)	2.8 (1.8–4.0)
Placental Growth Factor PLGF, pg/mL	30.6 (24.0–37.8)
Human chorionic gonadotropic hCG, IU/L	46.3 (30.2–70.2)
PAPPA Multiple of the Median (MoM)	1.2 (0.8, 1.7)
hCG MoM	1.0 (0.7, 1.6)
PLGF MoM	1.1 (0.9, 1.4)
*Birth delivery mode, n (%)*	
Spontaneous	362 (69.9.0%)
Induction	86 (16.6.5.0%)
Cesarean	68 (13.1%)
Gestational age at birth	39.3 ± 2.2
**Fetal Parameters**	
Crown–rump length	62.8 ± 6.4
Nuchal translucency thickness	1.9 ± 0.5
Fetal heart rate	160.3 ± 7.3
Ductus venosus pulsatility index	1.0 ± 0.2
Mean blood pressure, mmHg	85.8 ± 7.5
Mean uterine artery pulsatility index	1.6 ± 0.4
**Neonatal Parameters**	
Weight (g)	3248.9 ± 451
Length (cm)	49.9 ± 2
Cranial circumference (cm)	34.2 ± 1.5
Apgar score	9.8 ± 0.6

## Supplementary Materials

The following are available online at https://www.mdpi.com/1660-4601/17/23/9046/s1, Table S1: List of all collected variables.
